# Impact of back protectors on spinal injuries in alpine winter sports: a retrospective cohort study

**DOI:** 10.1007/s00068-025-02983-8

**Published:** 2025-10-28

**Authors:** Christian Tinner, Niklas Rutsch, Arne L. Ackermann, Sonja Häckel, Fabian Aregger, Jan Gewiess, Dominik A. Jakob, Sebastian F. Bigdon, Christoph E. Albers

**Affiliations:** 1 Department of Orthopaedic Surgery and Traumatology, Inselspital, Bern University Hospital, University of Bern, Freiburgstrasse 18 , Bern, 3010 Switzerland; 2 Graduate School for Health Sciences, University of Bern, Bern, Switzerland; 3 Department of Visceral Surgery, Lindenhofspital, Bern, Switzerland; 4 Department of Emergency Medicine, Inselspital, Bern University Hospital, University of Bern, Bern, Switzerland; 5 Department of Neurosurgery, Charité – Universitätsmedizin Berlin, Berlin, Germany

**Keywords:** Alpine sports, Spinal injuries, Protective devices, Injury severity score, Polytrauma, Safety gear

## Abstract

**Purpose:**

This retrospective cohort study investigated whether back protectors reduce the incidence and severity of spinal injuries in alpine winter sports, hypothesizing they may affect the type, location, and severity rather than fully prevent them.

**Methods:**

We retrospectively identified patients with alpine winter sports-related injuries admitted to our Level-1 trauma center (2017–2023). Patient and accident data were gathered via phone survey and chart review. Injuries were classified using the AO Spine system, and the impact of back protectors was analyzed using univariate and multivariate analysis in R.

**Results:**

Out of 1160 identified patients, 232 (81 spinal injuries, 151 non-spinal injuries) completed telephone follow-up (mean 52 months post-injury, SD 19.8). The presence of a spinal injury was not significantly associated with back protector use, but spinal injuries in protector users were more frequently treated conservatively, while non-users required operative treatment slightly more frequently (*p* = 0.13) and presented with neurological deficits (*p* = 0.008). After adjusting for confounders, there was no significant reduction in ISS scores. Moreover, wearing a back protector did not significantly impact the overall occurrence of polytrauma.

**Conclusion:**

Back protectors neither prevented spinal injuries nor provided beneficial protection in polytrauma cases among patients presenting to our level I trauma center.

**Supplementary Information:**

The online version contains supplementary material available at 10.1007/s00068-025-02983-8.

## Introduction

Over recent decades, alpine winter sports have surged in popularity, resulting in the proliferation of resorts and an increasing number of athletes [1]. Approximately 200 million individuals worldwide are active skiers, with an additional 70 million engaging in snowboarding [2, 3]. This rise in participation has been accompanied by a notable incidence of accidents [4]. Despite advancements in equipment, protective gear, and slope design aimed at mitigating risks, there has been a concurrent trend of higher speeds among athletes, leading to an elevated impact during accidents [2, 5–7]. The emergence of freestyle skiing and snowboarding, along with the provision of snowparks, has further elevated the risk of injuries [8–12].

Among younger, more physically active athletes, injuries typically stem from high-energy traumas [13]. In contrast, the aging population participating in winter sports exhibits distinct injury patterns, with lower extremity injuries being more prevalent and surpassing spinal injuries in frequency [7, 14, 15]. However, both multiple injuries and isolated spinal injuries—especially those that are life-altering—are common and impose a substantial burden on an individual’s quality of life, as well as a considerable socioeconomic burden on society [16]. Therefore, there is a need to improve safety and reduce the severity of injuries.

Back protectors constitute a critical safety component in alpine winter sports [17–19]. Engineered to offer supplementary protection for the spine and surrounding areas, particularly the thoracolumbar region, these devices are commonly utilized by athletes and enthusiasts in activities prone to high-impact collisions and falls. The primary advantage of back protectors lies in their capacity to absorb and disperse forces directly impacting the body during falls or collisions. Typically fabricated from specialized materials such as foam or impact-absorbing polymers, they effectively reduce the transmission of energy to the spine upon impact.

While the use of back protectors is recommended as a safety measure [20], ongoing research is necessary to evaluate their effectiveness in preventing spinal injuries. The aim of this study was to characterize spinal injuries related to alpine winter sports in relation to back protector use.

## Methods

### Study type

We conducted a retrospective cohort study to examine the influence of the use of back protectors on spinal injuries in alpine winter sports. The manuscript was prepared along the STROBE reporting guidelines for cohort studies [21].

### Study population

All medical records of adult patients (≥ 16 years) admitted to the emergency department within the specified time period were searched for the following keywords “ski, snowboard, carven, carving coupled with the Boolean operator “OR”. The medical emergency department report of every hit in our computerized database (Ecare, Turnhout, Belgium) was then manually screened to ensure that a winter sport injury was present.

Exclusion criteria were patients under the age of 16 years, as they are not routinely treated in the adult emergency department. Patients who did not consent to the use of their anonymized data or who refused were also excluded from the study.

To gain deeper insights into the characteristics of spinal injuries in alpine sports, we focused on patients with spinal injuries. In the next step, all individuals with a diagnosed spinal injury were identified. A matched non-spinal injury cohort (2:1 ratio) was then created based on age and sex using R, the R-Studio interface [22] and the “MatchIt“ [23] package, with the “nearest” method for enhancing comparability and reducing selection bias because of the suspected difference in cohort size and considering potential loss of follow-up data gaps.

### Variables and data sources

The review and approval by the regional ethics committee (Kantonale Ethikkommission Bern project-ID, 2023 − 01455) have been successfully completed. Based on the identified cohort, we conducted standardized phone interviews to gather details about each participants snow sports accident (questionnaire: Supplement 1). Informed consent was obtained at the beginning of each interview [24]. Contact was initiated via letter and phone. The interview focused on skill level, accident mechanism, and recovery during the follow-up period. To objectively assess proficiency, participants were asked about degree of difficulty of slopes they could navigate and the age at which they learned to ski or snowboard. The level was assigned to one of the difficulty categories of ski slopes in Switzerland: Blue for beginner slopes with a gentle incline, generally wide and well-groomed; Red for intermediate slopes, slightly steeper and requiring more experience; Black for advanced slopes with the steepest and most challenging parts, where expert skills are needed; and Yellow for unprepared, extremely steep, and freeride slopes. The majority demonstrated an advanced proficiency level, with most having started winter sports in childhood and mastering all slopes, including off-piste terrain.

All participant cases with spinal injuries were retrospectively reviewed by three spine surgeons with intermediate level of experience, two of them with 4 years of expertise (C.T. and F.A.), and one surgeon with over 5 years of experience (S.F.B.), who also served as a tiebreaker. Injuries were classified using the AO Spine classification system (Type A, Compression fractures, type B, hyperflexion or -extension fractures and type C, rotational or dislocated fractures) [25–28]. We allocated the level of spinal injury to one of the following regions: Cervical spine: C0-C7, thoracic spine: Th1-Th10, thoracolumbar spine: Th11-L2, lumbosacral spine: L3-S3. The Injury Severity Score (ISS) and Abbreviated Injury Scale (AIS) were obtained from the emergency department’s patient files and the swiss trauma registry.

We used the modified Tegner Activity Scale to evaluate patients’ activity levels before and after the accident. This scale was initially adapted by Reinke et al. for spine patients undergoing cervical disc arthroplasty implantation [29], the scale was later applied to individuals following 360-degree thoracolumbar fusion [30]. This standardized tool assesses functional capacity and physical activity levels, allowing for a structured comparison of pre- and post-injury activity in patients with spinal conditions.

### Statistical methods

All graphing and statistical analysis were performed in RStudio [22] (Version 4.3.2.), employing the package “tidyverse” [31], “tableone” [32], and “pscl” [33] for regression analysis. In the analysis of continuous variables, we employed parametric t-tests for normal distributed variables. For categorical variables, χ^2^ test were utilized to assess the association between the groups (injured while wearing a back protector vs. injured without wearing a back protector).

We used Fisher’s exact test to compare surgery rates between protector wearers and non-wearers. The risk ratio (RR) with a 95% confidence interval was calculated to quantify the association.

We evaluated the impact of wearing a spine protector on the likelihood of sustaining a spinal injury using a 2 × 2 table and χ^2^ testing. To account for potential confounders, a multivariable linear regression model was constructed using the log-transformed ISS as the dependent variable. The model adjusted for age, sex, type of sport (skiing vs. snowboarding), accident location (on vs. off piste), involvement of other persons, (self-) estimated speed, helmet use, self-assessed skill level, and self-assessed technical skiing ability [34].

## Results

**Fig. 1:**
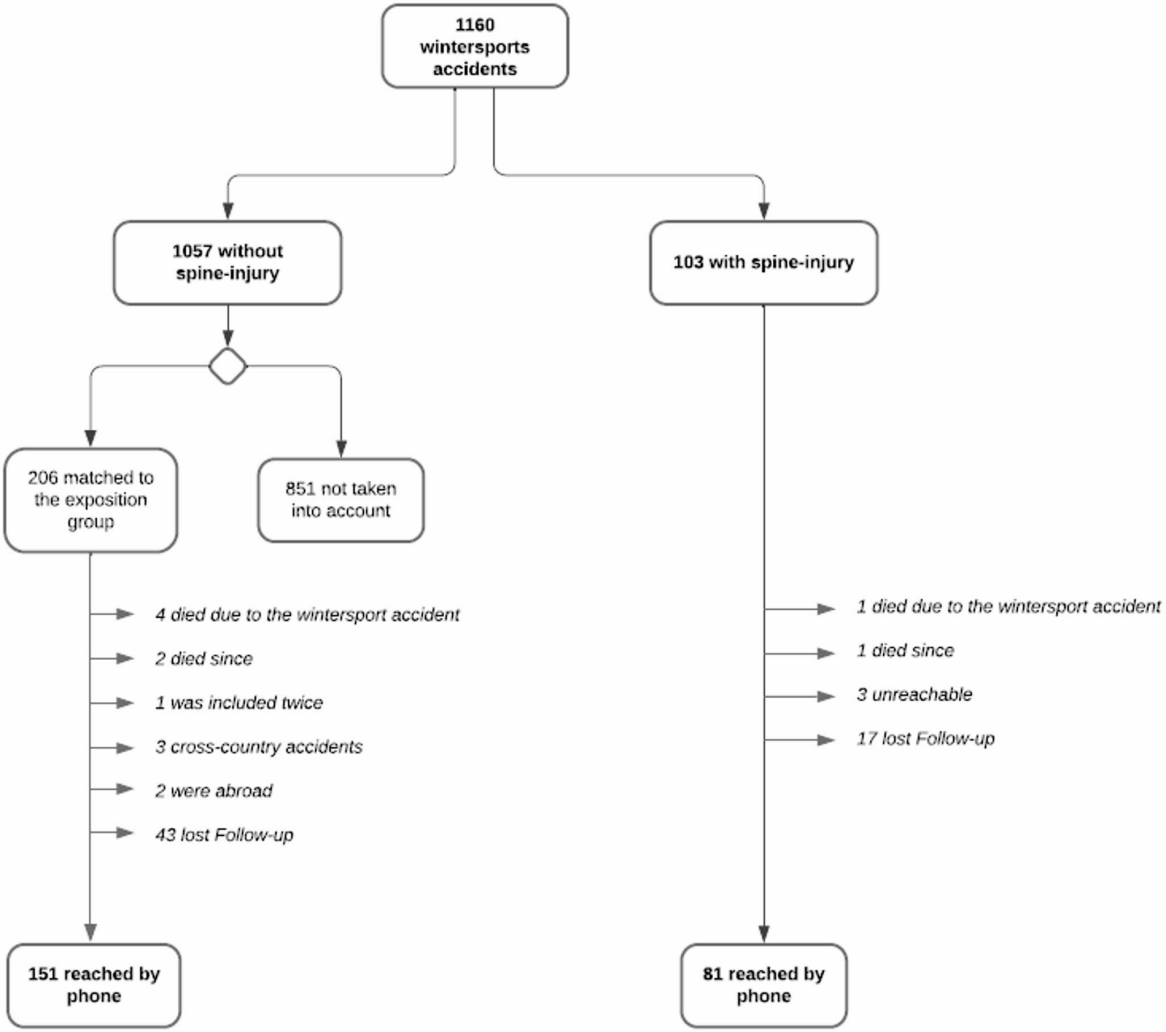
Flowchart depicting the selection process for patients with and without spinal injuries among 1160 winter sports-related accident cases between 2017 and 2023

Out of 1160 winter sports-related accident cases, 103 patients sustained spinal injuries (8.6%). From the non-spinal injury group, 206 patients (19.3%) were matched as controls based on age and sex. Follow-up rates were high for both groups, with 78.6% of the spinal injury group (*n* = 81) and 73.3% (*n* = 151) of the control group completing telephone follow-ups (Fig. 1).

The mean follow-up period was 52 months after the accident (range 22.1–87.16 months, SD = 19.8 months). Matching was successful, ensuring that the injury and control groups were comparable in terms of baseline characteristics. The study involved more than five times more men than women. Sex ratio did not differ between the groups (% female, control: 17.2% vs. spinal injury: 18.5%), nor did the location of the accident. Participants were highly physically active, with only 4% reporting no regular physical activity. The majority engaged in sports activity three or more times per week (Tegner 3–4, 67,7% vs. 64,8%) (Table 1). Activity levels were assessed using the modified Tegner activity score. Among patients with spinal injuries, 79% (*n* = 64) had a score between three to five, indicating moderate activities such as running two to four time per week, however, overall, there was no significant difference between the mean Tegner scores (*p* = 0.229).

**Table 1:** Population characteristics. Tegner activity levels: 1–2 (sedentary work), 3–4 (moderate activity), 5–6 (recreational), 7–10 (competitive)

Variable	No spinal injury	Spinal Injury
n	151	81
Female n (%)	26 (17.2)	15 (18.5)
Back Protector use n (%)	32 (21.2)	16 (19.8)
Age mean (SD)	45.97 (18.11)	45.28 (18.25)
Site of Injury n (%) On-Piste Snowpark Off-Piste	123 (82.0) 8 (5.3) 19 (12.7)	56 (71.8) 4 (5.1) 18 (23.1)
Activity n (%) Ski Snowboard Other	128 (84.8) 21 (13.9) 2 (1.3)	70 (86.4) 9 (11.1) 2 (2.5)
Perceived Skill Level n (%) Very good Good Intermediate Beginner	73 (49.3) 50 (33.8) 22 (14.9) 3 (2.0)	42 (54.5) 27 (35.1) 6 (7.8) 2 (2.6)
Tegner Score mean (SD) Levels n (%) 2 3 4 5 6 7 8 9	2.95 (1.12) 4 (3.0) 52 (39.1) 38 (28.6) 29 (21.8) 7 (5.3) 1 (0.8) 2 (1.5) 0 (0.0)	3.15 (1.14) 1 (1.4) 21 (29.6) 25 (35.2) 18 (25.4) 4 (5.6) 1 (1.4) 0 (0.0) 1 (1.4)

The majority of those who sustained spinal injuries rated themselves as very good to good skiers or snowboarders (89%).

Most accidents involving spinal injuries happened on the secured slopes (Table 1). Most patients (55.6%) reported that they were subjectively fast or very fast during their accident. Most accidents (86.0%) were self-caused and without anyone else involving. Paradoxically, despite these statistics, 77.8% (*n* = 63) of the participants with spinal injuries claimed that they were not passing beyond their skill level at the time of their accident.

### Distribution of spinal injuries in winter sports

**Fig. 2:**
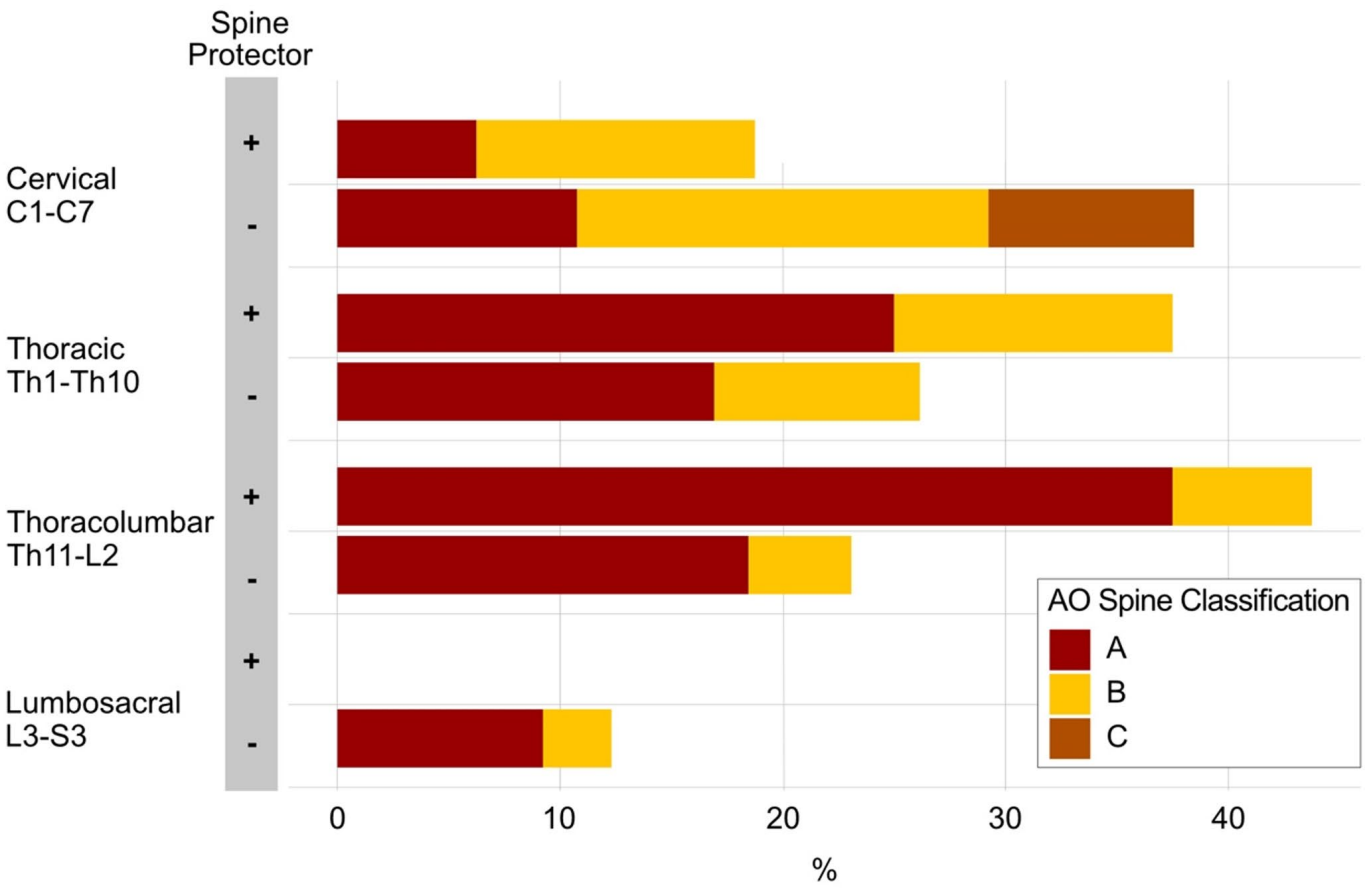
Spinal injuries in alpine winter sports. This depicts the distribution of spinal injuries dependent on the use of a back protector at the time of injury. Injury severity is represented by the color-coded AO spine classification system, according to the legend

Fractures were distributed throughout the entire spine, with the highest incidence observed in the thoracolumbar region. The most frequently affected vertebra was L1 (14.8%). AO fracture types A and B were the most common, comprising 47 (A, 58.0%) and 28 (B, 34.6%) cases, respectively, followed by type C with 6 (C, 7.4%) cases (Fig. 2).

### Influence of back protectors on spinal injuries

Athletes who had a back protector at the time of injury were significantly younger (age in years, mean (SD): no protector 48.88 (17.65) vs. protector 33.65 (14.60), *p* < 0.001). Significantly more snowboarders were found in the group wearing a back protector (no protector 7.1% vs. protector 35.4%, *p* < 0.001). The perceived skill level was not significantly associated with the use of a back protector. Notably, patients who wore back protectors at the time of the accident were significantly more active according to the modified Tegner activity score compared to those who did not wear a back protector (Tegner mean (SD); no protector 2.83 (0.95) vs. protector 3.71 (1.42); *p* < 0.001). However, athletes that injured themselves in a snow park were more likely to wear a back protector during the time of injury (*p* = 0.03). Suffering a spinal injury was not significantly dependent on the use of a back protector in univariate analysis (chi^2^ = 0.008, *p* = 0.93). The distribution of spinal injuries was not dependent on the use of a spine protector (*p* = 0.223, Fig. 2). Patients who did not wear a back protector at the time of injury (*n* = 65) primarily suffered from cervical spine injuries (38.5%) followed by thoracic (26.2%) and thoraco-lumbar (23.1%) injuries (Fig. 2). The severity of the spinal injury (as classified by the AO spine classification system) did not differ between the groups (*p* = 0.511). Spinal injuries in back protector wearers (*n* = 16) mostly affected the thoraco-lumbar region (43.8%) and the thoracic spine (37.5%). However, the occurrence of neurological deficits was significantly lower in protector wearers compared to non-wearers (0% vs. 17.7%, *p* = 0.008).

### Spine surgery following spinal injury

Of the 81 athletes that suffered a spinal injury, 16 (19.8%) wore a back protector at the time of injury. Surgical intervention was required in 4 of 16 (25%) protector wearers and 32 of 65 (49.2%) non-wearers. The risk of requiring surgery was lower among protector wearers (RR = 0.51, 95% CI: 0.21–1.23), though this difference did not reach statistical significance (*p* = 0.13). In athletes who wore a protector, all surgeries were performed using a percutaneous posterior approach, and none had neurological deficits. Among non-wearers, a wider range of surgical strategies was applied, including posterior open and 360° procedures, with some cases involving decompression. Eight patients (17.7%) suffered injuries of the neurologic structures with neurologic compromise.

### Wearing a spine protector does not reduce the injury severity score

Wearing a spine protector was not associated with a significant reduction in injury severity. In univariate analysis, there was no statistically significant difference in injury severity score (ISS) between individuals who wore a spine protector and those who did not (Supplement 2, Table 1). After adjusting for potential confounding variables, wearing a spine protector remained not significantly associated with injury severity (*p* = 0.51). Higher injury severity was significantly associated with increasing age (, *p* < 0.001), off-piste accidents (*p* = 0.04), and high self-reported speed at the time of the accident (*p* < 0.001) (Supplement 2, Table 2).

*Return to sports and impact on the health-related quality of life following alpine winter sports spinal injuries*.

**Fig. 3:**
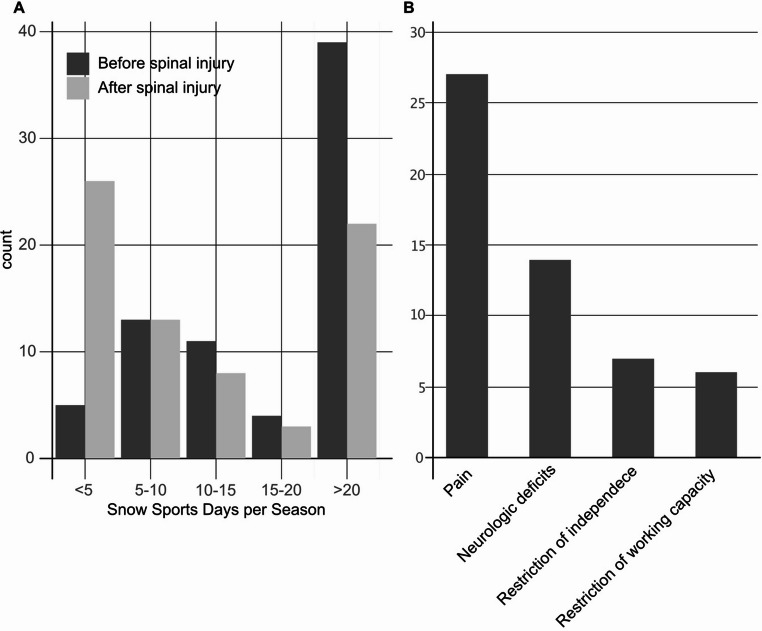
Quality of life (**A**) Number of snow sports days per season before and after the accident. (**B**) Residual symptoms affecting the patients’ quality of life during follow-up in patients with injuries related alpine winter sports accidents

Primary, we assessed the return to alpine sports after the spinal injury. While most athletes (*n* = 39, 48.1%) reported > 20 days/season before their accident, the predominant category after the accident was < 5 five days/season. The majority (*n* = 26, 32.1%) of all respondents have stopped doing winter sports since the accident (Fig. 3A).

Following the injury, 60.5% (*n* = 49) of individuals indicated subsequent impacts on their quality of life during our follow-up period. These were mainly characterized by pain (33.3% *n* = 27) followed by neurological impairment and restriction in their daily and working life activities (Fig. 3B). The return to the level of activity was not significantly associated with protector use and level of activity prior to injury.

## Discussion

In recent years, the use of helmets in alpine winter sports has significantly increased. But the use of helmets in alpine winter sports is still a topic of controversial discussion [13, 35, 36]. On one hand, it has been shown that helmets can reduce the severity of head and midface injuries [37]. On the other hand, increased risk-taking behavior and thus the likelihood of accidents may also rise [38]. Considerable research has been conducted to investigate these correlations in the context of helmet use in alpine winter sports and for cyclists. However, the situation is different when it comes to personal protective equipment for preventing spinal injuries, such as spine protectors.

Although numerous studies have examined risk prevention related to helmet use, the role of back protectors in winter sports has not been extensively researched.

Despite the common use of back protectors, designed to absorb and distribute impact forces, our findings suggest, that back protectors do not significantly influence the overall distribution of spinal injuries, nor do they reduce overall injury severity (ISS Score).

### Effectiveness of back protectors

Our data show a statistically non-significant trend toward a possible reduction in spine injury severity with lower rate of neurological involvement and fewer spinal injuries requiring surgery among individuals who wore back protectors during their accidents. This suggests that back protectors could be effective in mitigating the severity of injuries in high-impact scenarios. Within a meta-analysis, Ekmejan et al. showed possible positive effects of hard shell back protectors in a motorcyclist population but no specific benefit on spinal injuries [39]. Our findings are in line with it evaluating the impact on different protection measures in alpine winter sports. The materials used in back protectors, such as foam and impact-absorbing polymers, are likely contributing factors to this reduction by dissipating the forces experienced during falls or collisions [19, 40]. However, this protective effect did not extend to preventing the occurrence of spinal injuries, as the incidence rates were similar between users and non-users of back protectors.

Previous studies have similarly highlighted the protective benefits of equipment like helmets in reducing head injuries in winter sports, suggesting that specialized protective gear can significantly enhance safety in high-risk environments [35, 37, 41–43]. But interestingly despite of increasing use of helmets, Sulheim et al. showed an unexpected reduction in the protective effect of a skiing helmet. This may be due to new skiing trends and riskier behavior in the alpine resorts [36].

Snowboarders, beginners and children seem to have an increased overall injury risk on the slopes [13] and beginners are overrepresented among injured powder skiers off-piste [44]. In polytraumatized patients the ISS in adolescents was reported to be higher than in other age groups and alpine skiers suffered more severe injuries than snowboarders [45]. Brooks et al. say that more injuries occur among self-rated intermediates and experts than beginners [46]. In summary young intermediate to expert alpine skiers and snow park snowboarders lead to more severe injuries due to higher speed and more dangerous tricks in the snow park resulting in high impact forces during a crash. The findings of our study reinforce the importance of using back protectors, particularly in these scenarios involving high-speed impacts, where high impacts can be absorbed by protectors and may reduce the severity in case of a spinal injury.

### Spinal injury patterns

Skiers and snowboarders evaluated at the hospital are equally likely to have sustained spinal injuries, as confirmed in our data. Additionally, athletes in both sports have an increased incidence of SCI with cervical spine trauma [47].

The thoracolumbar spine is affected in almost two thirds of all traumatic spinal injuries. Our cohort also showed a high incidence in thoracolumbar junction injuries, however, other segments of the spine were involved more often than in the general trauma population. Our study found no significant difference in the distribution of spinal injury locations between those who wore back protectors and those who did not. Both groups experienced a high incidence of thoracic and thoraco-lumbar injuries, with a notable proportion of cervical spine injuries in the non-protector group.

It is also quite astonishing that patients without back protectors more frequently and with more severe injuries suffered from cervical spine injuries. This difference is statistically not significant but clearly visible (*p* = 0.511). We did not expect this effect and currently have no explanation for it. The cervical spine is naturally very susceptible to injuries due to its exposure to large forces during high-energy falls. We feared that the more rigid stabilization provided by the back protector might result in greater force being exerted on the cervical spine, potentially leading to more severe injuries. However, this hypothesis was not confirmed by our findings.

Different protector models typically cover the thoracic and lumbar spine region and protect it from direct impact. As shown in a biomechanical study, most forces in typical backwards falls, especially in snowboarding with direct contusion and hyperflexion, result in mid-thoracic injuries [17]. However, most of our reported fractures result from axial compression (Type A fractures), followed by hyperflexion or hyperextension (type B fractures) and rotational forces (type C fractures). Although the biomechanical study shows back protectors may protect from direct contusion and hyperflexion [19, 48], our results demonstrate that they do not offer protection against other common spinal injury mechanisms. Moreover, they may not provide comprehensive protection across all spinal regions, particularly the cervical area, which remains vulnerable. This aligns with existing literature emphasizing the need for additional protective measures, such as helmets, to safeguard the cervical spine.

### Demographic and behavioral insights

Our findings revealed that athletes who wore back protectors were significantly younger and more likely to be snowboarders. This demographic might be associated with higher-risk behaviors and a propensity for engaging in freestyle activities, which could explain the more frequent back protector use [19, 47]. Most spinal injuries occurred on secured slopes, and most accidents were self-caused, highlighting the importance of personal protective equipment regardless of the perceived safety of the environment.

These observations are consistent with previous studies that have noted the increased risk-taking behavior among younger athletes and snowboarders. The self-reported high skill level among injured athletes suggests that even experienced athletes are not immune to accidents especially with high impact, underscoring the need for widespread use of protective gear.

### Impact on quality of life and activity levels

The study underscores the long-term impact of spinal injuries on quality of life and activity levels. Although most injuries only were AO Spine type A or B, a significant portion of respondents reported ongoing pain, neurological impairment, and restrictions in daily activities, which adversely affected their return to winter sports. Moreover, patients with spinal cord injury cause substantial socioeconomic costs [16]. Therefore, prevention and education as well as use of back protectors may help in mitigating this condition.

In general, young patients with thoracolumbar burst fractures treated with 360° fusion show higher rate of return to sports compared to our cohort [49]. Despite the reduced severity of injuries with back protector use, the overall impact on athletes’ post-accident sports engagement remained substantial, with many reducing their activity levels or ceasing participation altogether. This points to the need for comprehensive rehabilitation programs and ongoing support for injured individuals to facilitate their return to sport and regular activities.

### Public involvement: expert skiers’ perspectives on back protectors

The use of back protectors is also a topic of discussion among professional athletes in alpine winter sports. In alpine skiing, airbag vests have recently become mandatory by International Ski and Snowboard Federation (FIS), back protectors have been already mandatory since 2006 in speed races; however, in freestyle skiing and snowboarding, helmets remain the only obligatory protective equipment. We interviewed 17 active professional Swiss winter sports athletes, including 11 alpine skiers, two freestyle skiers, and four freestyle snowboarders. Twelve out of the 17 athletes (71%) reported using a back protector, with half of them wearing it consistently and the rest using it during training and competitions. While all alpine skiers wear an airbag vest due to the mandate, none of the athletes reported receiving recommendations from their coaches regarding the use of a back protector. Only two athletes believed that back protectors specifically prevent spinal injuries. Six athletes felt that back protectors offer protection against direct impacts, such as collisions with rails, hard slopes of safety nets. Another five athletes noted that back protectors provide general protection and enhance trunk stability. Two freestyle athletes mentioned that back protectors can limit their range of motion. However, Andri Ragettli, with 31 World Cup podium finishes, including 12 victories, 5 crystal globes, and 3 X-Games gold medals, always wears a back protector. He believes that it helps prevent injuries from direct impacts with rails, icy landings, and general impacts to the trunk, as well as burns from sliding on the snow during falls. Similarly, Marco Odermatt, one of the world’s most successful alpine skiers and an Olympic gold medalist with multiple overall World Cup wins and three World Championship titles, stated that he always wears both a back protector and an airbag vest and started to wear an airbag vest as one of the first alpine skiers in 2018. He emphasized that the back protector with the airbag vest provide him with a general sense of safety.

### Strength and limitations and future research

A significant strength of this study is its comprehensive data collection from a well-defined patient cohort of the largest level one trauma center in Switzerland over an extended period. Additionally, the use of the AO Spine classification system for detailed and standardized injury assessment, along with advanced statistical methods like adjusted regression models, adds rigor and depth to the analysis. Focusing on polytraumatized patients provides valuable insights into a critical and often overlooked aspect of spinal injury research in snow sports.

However, the study faces limitations, including potential selection bias, as it only includes patients admitted to a level one trauma center. The study setting does not allow us to determine whether spine protectors prevent spinal injuries, since only patients who sustained injuries and presented to the emergency department were included. Therefore, the study only permits comparisons between injured patients with and without back protectors. The retrospective design and reliance on historical medical records may introduce recall bias and affect the accuracy of reported incidents and outcomes. The absence of randomization might allow for confounding factors, such as more cautious individuals who are more likely to wear protectors and less likely to engage in risky behaviors. Additionally, while we adjusted for various confounders, other unmeasured variables could influence the results.

## Conclusion

In conclusion, back protectors in alpine winter sports did not influence the incidence or distribution of spinal injuries among patients presenting to our level I trauma center. Furthermore, there was no significant difference in injury severity or in the occurence of polytrauma between patients with and without back protectors. Future studies should focus on the biomechanical effectiveness of different protector designs, emphasizing injury mechanisms and real-world efficacy across various alpine sports contexts.

## Supplementary Information

Below is the link to the electronic supplementary material.

Supplementary Material 1 (DOCX 15.8 KB)

Supplementary Material 2 (DOCX 29.0 KB)

## Data Availability

Data are available upon reasonable request from the corresponding author.
