# Supplementary material for: Impact of back protectors on spinal injuries in alpine winter sports: a retrospective cohort study
Source: Eur J Trauma Emerg Surg. 2025 Oct 28;51(1):309. doi: 10.1007/s00068-025-02983-8 (PMC12568888; doi:10.1007/s00068-025-02983-8)
Supplement: Supplementary file 1 — Supplementary Material 1 (DOCX 15.8 KB) [file 68_2025_2983_MOESM1_ESM.docx]

**Fragebogen**

*Einfluss von Rückenprotektoren auf Wirbelkörperfrakturen bei Wintersportunfällen*

1. Art des Unfalls
   1. Ski Unfall
   2. Snowboard Unfall
   3. Anderes__________________________
2. Trugen sie beim Skiunfall einen Rückenprotektor
   1. Ja
   2. Nein
   3. Wenn ja, welcher?
      1. ___________________________________
   4. Wenn ja, Art des Rückenpanzers
      1. Gilet mit Reisverschluss
      2. Rucksack
      3. Einfaches Modell
   5. Wenn ja, mussten sie nach dem Unfall einen neuen besorgen oder war er noch brauchbar
      1. Neu
      2. brauchbar
   6. Wenn ja, hatten sie das Gefühl ohne ihn wäre es noch schlimmer gekommen
      1. Ja
      2. Nein
   7. Wenn ja, hatten sie dadurch das Gefühl von zusätzlicher Sicherheit und sind deshalb über ihre Grenzen gegangen?
      1. Ja
      2. nein
   8. Wenn nein, tragen sie seit dem Unfall einen Rückenprotektor
      1. Ja
      2. nein
   9. Wenn nein, denken sie das Tragen eines Rückenprotektor hätte die Folgen des Unfall gelindert oder verhindert?
      1. Ja
      2. Nein
3. Unfallort
   1. Piste
   2. Park
   3. Off-Piste
4. Unfallhergang
   1. Weitere Personen involviert
   2. Selbstverschuldet/fremdverschuldet
   3. Tempo
      1. Langsam
      2. Schnell
      3. Sehr schnell
   4. Unfallvorgang
      1. Direkter Anprall
      2. Flexion (Beugung, Einknicken)
      3. Extension (Überstreckung)
      4. Mehrfaches Überschlagen
      5. ___________________
   5. Haben sie einen Helm getragen
      1. Ja
      2. Nein
5. Wie hat sich ihr Verhalten seit dem Unfall auf den Skiern verändert
   1. Gar nicht
   2. Ich trage seither einen Rückenprotektor
   3. Ich bin vorsichtiger geworden
   4. Ich meide gewisse Abfahrten
   5. Ich fahre seither kein Ski mehr
6. Wieviele Schneetage pro Saison nach Unfall vor Unfall
   - 1. < 5. < 5
     2. 5-10 5-10
     3. 10-15 10-15
     4. 15-20 15-20
     5. 20 > 20 >
7. Haben sie das Gefühl sie haben sich bei ihrem Unfall über dem Limit ihres Könnens bewegt?
   1. Ja
   2. nein
8. Skill-Niveau
   1. Subjektives Level vor dem Unfall
      1. Sehr gut
      2. Gut
      3. Mittel
      4. Anfänger
   2. Skifahren seit
      1. Kindheit (0-12)
      2. Jugend (12-20)
      3. Frühes Erwachsenenalter (bis 30)
      4. Spätes Erwachsenenalter (ab 30)
   3. Welche Pisten
      1. rot, blau
      2. + Schwarz
      3. + off-piste
9. Subjektiv eingeschätztes Können vor dem Unfall
   1. Anfänger
   2. Immer Rutschend
   3. Rutschend im steilen, carving im flachen
   4. Immer Carvend
   5. Sicher auf und neben der Piste
10. Treiben sie sonst Sport (vor Unfall)
    1. Ja
    2. Nein
    3. Wenn ja, was für Sport
       1. ____________________________________________________
    4. Wenn ja, wie oft mal in der Woche treiben sie Sport
       1. Mehr als 3x pro Woche
       2. 1x pro Woche
       3. 1-2x im Monat Tegner:________
       4. Weniger
11. Hatten sie seit ihrem Unfall Einschränkungen aufgrund ihrer Wirbelkörperfraktur?
    1. Wenn ja welche
       1. Schmerzen
       2. Neurologie
       3. Selbstständigkeit
       4. Arbeitstätigkeit
       5. __________________
    2. nein
