# Supplementary material for: Impact of back protectors on spinal injuries in alpine winter sports: a retrospective cohort study
Source: Eur J Trauma Emerg Surg. 2025 Oct 28;51(1):309. doi: 10.1007/s00068-025-02983-8 (PMC12568888; doi:10.1007/s00068-025-02983-8)
Supplement: Supplementary file 2 — Supplementary Material 2 (DOCX 29.0 KB) [file 68_2025_2983_MOESM2_ESM.docx]

**Supplement 2**

| Variable | No protector | Protector | p |
| --- | --- | --- | --- |
| n | 184 | 48 |  |
| Age (mean (SD)) | 48.88 (17.65) | 33.65 (14.60) | <0.001 |
| Female (%) | 33 (17.9) | 8 ( 16.7) | 1.000 |
| Type of sport |  |  | <0.001 |
| Ski | 168 (91.3) | 30 ( 62.5) |  |
| Snowboard | 13 ( 7.1) | 17 ( 35.4) |  |
| Other | 3 ( 1.6) | 1 (  2.1) |  |
| Site of accident |  |  | 0.41 |
| On-Piste | 144 (80.0) | 35 ( 72.9) |  |
| Park | 6 ( 3.3) | 6 ( 12.5) |  |
| Off-Piste | 30 (16.7) | 7 ( 14.6) |  |
| Speed (%) |  |  | 0.136 |
| Slow | 94 (53.7) | 23 ( 47.9) |  |
| Medium | 75 (42.9) | 20 ( 41.7) |  |
| Fast | 6 ( 3.4) | 5 ( 10.4) |  |
| Mechanism (%) |  |  | 0.100 |
| Direct Contact | 49 (81.7) | 8 ( 53.3) |  |
| Flexion | 1 ( 1.7) | 2 ( 13.3) |  |
| Extension | 1 ( 1.7) | 0 (  0.0) |  |
| Roll over | 7 (11.7) | 4 ( 26.7) |  |
| Direct hit and roll over | 2 ( 3.3) | 1 (  6.7) |  |
| No helmet (%) | 28 (15.4) | 0 (  0.0) | 0.008 |
| Ski days before injury per season (%) |  |  | 0.885 |
| <5 | 4 ( 7.1) | 1 (  6.2) |  |
| 5-10 | 9 (16.1) | 4 ( 25.0) |  |
| 10-15 | 8 (14.3) | 3 ( 18.8) |  |
| 15-20 | 3 ( 5.4) | 1 (  6.2) |  |
| >20 | 32 (57.1) | 7 ( 43.8) |  |
| Ski days after injury (%) |  |  | 0.801 |
| <5 | 21 (37.5) | 5 ( 31.2) |  |
| 5-10 | 10 (17.9) | 3 ( 18.8) |  |
| 10-15 | 5 ( 8.9) | 3 ( 18.8) |  |
| 15-20 | 2 ( 3.6) | 1 (  6.2) |  |
| >20 | 18 (32.1) | 4 ( 25.0) |  |
| Did not feel over the limit (%) | 160 (88.9) | 40 ( 85.1) | 0.645 |
| Subjective skill (%) |  |  | 0.729 |
| Very good | 88 (49.4) | 27 ( 57.4) |  |
| Good | 62 (34.8) | 15 ( 31.9) |  |
| Intermediate | 24 (13.5) | 4 (  8.5) |  |
| Beginner | 4 ( 2.2) | 1 (  2.1) |  |
| Skiing since (%) |  |  | 0.557 |
| Childhood | 145 (83.3) | 38 ( 80.9) |  |
| Youth | 20 (11.5) | 8 ( 17.0) |  |
| Early adulthood | 5 ( 2.9) | 1 (  2.1) |  |
| Late adulthood | 4 ( 2.3) | 0 (  0.0) |  |
| Which pistes are common (%) |  |  | 0.963 |
| Blue/Red | 13 ( 7.3) | 3 (  6.4) |  |
| Black | 59 (33.0) | 15 ( 31.9) |  |
| Off-Piste | 107 (59.8) | 29 ( 61.7) |  |
| No frequent physical activity (%) | 16 ( 9.1) | 2 (  4.2) | 0.411 |
| Times physical activity per week (%) |  |  | 0.829 |
| >3x | 87 (55.1) | 27 ( 60.0) |  |
| 1x | 68 (43.0) | 17 ( 37.8) |  |
| 1-2/month | 2 ( 1.3) | 1 (  2.2) |  |
| less | 1 ( 0.6) | 0 (  0.0) |  |
| ISS (median [IQR]) | 4.00 [4.00, 9.25] | 4.00 [2.00, 8.00] | 0.087 |
| Ais head (median [IQR]) | 2.00 [1.00, 2.00] | 2.00 [1.00, 2.00] | 0.938 |
| Ais face (median [IQR]) | 0.00 [0.00, 1.75] | 0.00 [0.00, 2.00] | 0.707 |
| Ais neck (median [IQR]) | 0.00 [0.00, 2.00] | 1.00 [0.00, 2.00] | 0.409 |
| Ais thorax (median [IQR]) | 1.00 [0.00, 3.00] | 2.50 [1.25, 3.00] | 0.149 |
| Ais abdomen (median [IQR]) | 0.00 [0.00, 2.00] | 0.50 [0.00, 1.75] | 0.648 |
| Ais spine (median [IQR]) | 0.00 [0.00, 1.00] | 0.00 [0.00, 1.00] | 0.966 |
| Ais upper extremities (median [IQR]) | 2.00 [0.00, 2.00] | 2.00 [1.00, 2.00] | 0.474 |
| Ais lower extremities (median [IQR]) | 1.00 [0.00, 2.00] | 1.00 [1.00, 2.00] | 1.000 |
| Ais external (median [IQR]) | 0.00 [0.00, 0.00] | 0.00 [0.00, 0.00] | 0.613 |

**Table 1 Univariate analysis protector wearers vs. no protector wearers**

**Supplement 2**

**Table 2 Linear regression model for the ISS adjusted for confounding variables**

|  | Estimate | Std. Error | t value | Pr(>\|t\|) |  |
| --- | --- | --- | --- | --- | --- |
| (Intercept) | 1,345 | 0,910 | 1,478 | 0,141 |  |
| Protector = Yes | -0,099 | 0,148 | -0,665 | 0,507 |  |
| Age | 0,013 | 0,004 | 3,678 | 0,000 | *** |
| Male | -0,042 | 0,152 | -0,275 | 0,783 |  |
| Type of sport |  |  |  |  |  |
| Snowboard | -0,164 | 0,178 | -0,919 | 0,359 |  |
| Other | -0,377 | 0,767 | -0,492 | 0,623 |  |
| Site of accident |  |  |  |  |  |
| Park | 0,191 | 0,249 | 0,768 | 0,443 |  |
| Off-Piste | 0,339 | 0,163 | 2,071 | 0,040 | * |
| Persons involved |  |  |  |  |  |
| Self-induced | 0,144 | 0,312 | 0,461 | 0,645 |  |
| Caused by others | -1,312 | 0,763 | -1,719 | 0,087 | . |
| Self induced with others  involved | -0,547 | 0,193 | -2,836 | 0,005 | ** |
| Self-estimated speed |  |  |  |  |  |
| Fast | 0,428 | 0,121 | 3,535 | 0,001 | *** |
| Very fast | 0,381 | 0,249 | 1,526 | 0,129 |  |
| No helmet | -0,058 | 0,171 | -0,336 | 0,737 |  |
| Skill level |  |  |  |  |  |
| Good | 0,118 | 0,119 | 0,992 | 0,323 |  |
| Intermediate | 0,089 | 0,198 | 0,448 | 0,654 |  |
| Beginner | 0,754 | 0,812 | 0,929 | 0,354 |  |
| Carving skills |  |  |  |  |  |
| Just sliding | -0,443 | 0,855 | -0,518 | 0,605 |  |
| Sliding+Carving | -0,365 | 0,891 | -0,410 | 0,683 |  |
| Carving | -0,345 | 0,893 | -0,386 | 0,700 |  |
| Safe skiing in all conditions | -0,277 | 0,882 | -0,313 | 0,754 |  |

Multiple R-squared: 0.1921

Adjusted R-squared: 0.1075

F-statistic: 2.271 on 20 and 191 DF

p-value: 0.002295
